# Sea surface temperature variability and ischemic heart disease outcomes among older adults

**DOI:** 10.1038/s41598-021-83062-x

**Published:** 2021-02-09

**Authors:** Haris Majeed, Rahim Moineddin, Gillian L. Booth

**Affiliations:** 1 Institute of Medical Science, Temerty Faculty of Medicine, University of Toronto, 1 King’s College Circle, Toronto, ON M5S 1A8 Canada; 2 MAP Centre for Urban Health Solutions, Li Ka Shing Knowledge Institute of St Michael’s Hospital, Unity Health Toronto, 209 Victoria Street, Toronto, ON M5B 1T8 Canada; 3 Department of Family and Community Medicine, Temerty Faculty of Medicine, University of Toronto, 500 University Avenue, Toronto, ON M5G 1V7 Canada; 4 Institute of Health Policy, Management and Evaluation, Dalla Lana School of Public Heath, University of Toronto, 155 College Street, Toronto, ON M5T 1P8 Canada; 5 Department of Medicine, Temerty Faculty of Medicine, University of Toronto, 1 King’s College Circle, Toronto, ON M5S 1A8 Canada

**Keywords:** Environmental impact, Risk factors, Physical oceanography, Climate-change impacts

## Abstract

Ischemic heart disease (IHD) is one of the leading causes of death worldwide. While extreme summer surface air temperatures are thought to be a risk factor for IHD, it is unclear whether large-scale climate patterns also influence this risk. This multi-national population-based study investigated the association between summer Pacific and Atlantic sea surface temperature (SST) variability and annual acute myocardial infarction (AMI) or IHD event rates among older adults residing in North America and the United Kingdom. Overall, a shift from cool to warm phase of the El Niño Southern Oscillation (ENSO) was associated with reduced AMI admissions in western Canada (adjusted rate ratio [RR] 0.89; 95% CI, 0.80–0.99), where this climate pattern predominatly forces below-normal cloud cover and precipitation during summertime, and increased AMI deaths in western United States (RR 1.09; 95% CI, 1.04–1.15), where it forces increased cloud cover and precipitation. Whereas, the Atlantic Multidecadal Oscillation (AMO) during a strong positive phase was associated with reduced AMI admissions in eastern Canada (RR 0.93; 95% CI, 0.87–0.98) and increased IHD mortality during summer months in the United Kingdom (RR 1.08; 95% CI, 1.03–1.14). These findings suggest that SST variability can be used to predict changes in cardiovascular event rates in regions that are susceptible.

## Introduction

Ischemic heart disease (IHD) is the leading cause of death worldwide and one of the common reasons for hospital admission in men and women^1,2^; driven in large part by episodes of acute myocardial infarction (AMI). Some literature suggests that extreme warm and cold surface air temperatures (SAT) are associated with an increased incidence of IHD events in northern countries^3–7^ especially among older adults who are vulnerable to such changes^8^, while other studies have not shown a significant relationship^9,10^. Numerous physiologic mechanisms have been proposed to explain these associations, ranging from cold-induced arterial vasoconstriction resulting in increased cardiac output and blood fibrinogen concentrations in cold SAT^7^ to intravascular volume depletion and dehydration during periods of extreme heat^11^, phenomena that have been linked to an increased risk of IHD events. Specifically, hot humid conditions causes a redirection of blood flow to the skin and loss of fluids from sweating, which can lead to tachycardia and even hypotension, particularly in older populations who have a diminished thirst response and may be on blood pressure medications that contribute to dehydration^12^. These processes, in turn, can precipitate cardiac ischemia due to reductions in coronary blood flow. Summer heat can also lead to sleep disturbances^13^, which in older populations can worsen glucose intolerance and cause disruptions in circadian rhythms^14^. Despite the surge of climate-health related research in the past decade^8^, few studies have examined the association between cardiovascular disease and large-scale climate patterns arising from natural climate variability^15–17^.

Natural climate variability is largely driven by the Earth’s systems such as the oceanosphere, which comprises more than 70% of the Earth’s surface area^18^. Consequently, climatological conditions in North America and the United Kingdom are dictated to a large extent by variability in Pacific and Atlantic sea surface temperatures (SST), through the El Niño Southern Oscillation (ENSO) and Atlantic Multidecadal Oscillation (AMO), respectively^19–26^. The ENSO’s two dominant phases are El Niño (warm) and La Niña (cool)^26^, where AMO has a positive (warm) and negative (cool) phase^23^. An El Niño event generally creates warmer than average conditions over western regions of North America^24,25^. The strong El Niño event of 2015–2016 also resulted in above-normal spring/summer rainfall anomalies in western United States, yielding regional infectious disease outbreaks^27^. Similarly, a positive (warm) phase of the AMO results in warmer than average conditions over eastern regions of North America and western Europe^23^. Atlantic SST variability is believed to be linked to the surge of European heatwave-related deaths in the summer of 2003^28^.

Interannual variability in SST-related climate patterns, such as ENSO and AMO impact air mass over land, leading to disruption in regional SAT, cloud cover, and precipitation anomalies^23,24^, which could directly or indirectly influence IHD outcomes. No studies have assessed for differential effects of ENSO/AMO on IHD event rates across regions where they cause divergent climatological conditions. Hence the objective of this study was to examine the association between interannual SST variability and IHD outcomes among older adults residing in North America and the United Kingdom from 2000–2017. While local climatological conditions may have greater effects, ENSO/AMO represent ‘upstream’ climate patterns which have a vital influence on local climate for large areas of North America, the United Kingdom, and other regions. At the same time, SST variability could be used to forecast the number of IHD events among older adults residing in susceptible areas; which is more difficult to do based on a single climatological variable. We focused on the ENSO and AMO resulting from Pacific and Atlantic SST variability, respectively, during summertime since the initiation of an ENSO event typically begins in boreal spring/summer^26^ and AMO has strong climatological impact over western Europe during summer^23^.

We hypothesized that interannual variability in summer ENSO would be significantly associated with AMI admission and mortality rates among older adults residing in western regions of United States and Canada, whereas interannual variability in summer AMO would be significantly associated with eastern regions of the United States and Canada, as well as the United Kingdom. Furthermore, we hypothesized that regional differences in climate caused by ENSO/AMO could lead to variable effects on IHD incidence and mortality. For example, regions that experience more cloud cover and precipitation in response to positive (warm) phases of ENSO/AMO could experience wetter/humid summers, which may increase the risk of ischemic events. These associations were investigated by combining national disease surveillance and climatological data to understand the influence of variability in oceanic climate patterns on IHD outcomes among older adults.

## Results

This study included data on more than 18,383,937 Americans, 2,951,913 Canadians, and 4,787,516‬ British citizens who were ≥ 65 years old for our study period. During the follow-up period, there were a total of 426,108 deaths following AMI in the United States, 349,470 AMI admissions in Canada, and 27,337 IHD deaths in the United Kingdom. There were notable declines in cardiovascular disease endpoints amongst older men and women in each of the countries over the period of study (Supplementary eTable 1). In addition, there were significant variations in event rates across regions. Within the United States, AMI mortality rates were highest in the South, whereas in Canada, the highest rates of admission for AMI were in the East.

**Figure 1:**
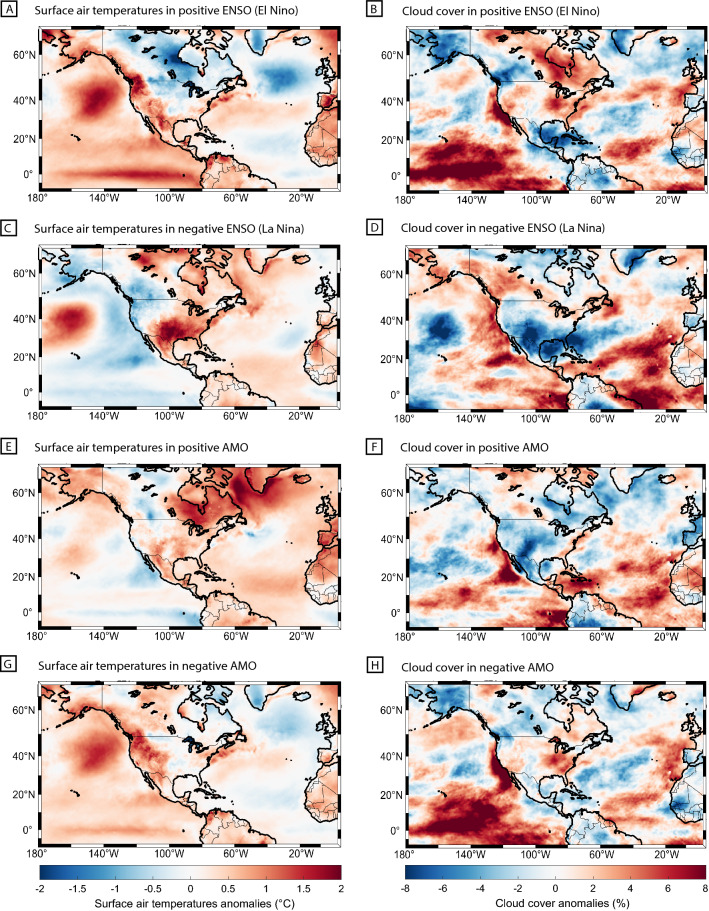
Summer ENSO and AMO Impact on Surface Air Temperatures and Cloud Cover. (**A**) Average summer surface air temperature (°C) and (**B**) cloud cover (%) anomalies during El Niño phases in 2002, 2009, 2015. (**C**) Average summer surface air temperature (°C) and (**D**) cloud cover (%) anomalies during La Niña phases in 2000, 2010, 2011. (**E**) Average summer surface air temperature (°C) and (**F**) cloud cover anomalies (%) during positive AMO phase in 2003, 2005, 2010. (**G**) Average summer surface air temperature (°C) and (**H**) cloud cover anomalies during negative AMO phase in 2002, 2013, 2015. Anomalies are based on the reference period of 1981–2010. The ERA5 dataset was used in MATLAB R2017b (https://www.mathworks.com/products/matlab.html).

Different climatological conditions have been demonstrated during certain phases of ENSO and AMO, with specific features depending on the affected region. As illustrated in Fig. 1, during summer El Niño events (warmer Pacific SST), SAT were on average ~ 1 °C warmer in western regions of North America (Fig. 1A), with increased cloud cover over the western United States, but reduced cloud cover over western Canada (Fig. 1B). During summer La Niña events (cooler Pacific SST), SAT were on average ~ 1 °C cooler over western regions of North America (Fig. 1C) and this was linked to increased cloud cover over western Canada, but reduced cloud cover over the United States (Fig. 1D). During a positive phase of AMO, the Northeast United States and East Canada experienced on average increase in SAT ~ 1 °C above the reference period (Fig. 1E), with reduced cloud cover (Fig. 1F). The opposite was found during a negative AMO where slightly cooler SAT (Fig. 1G) and increased cloud cover were observed over northeastern regions of North America (Fig. 1H). The general regional-specific climatological outlook during ENSO/AMO phases are summarized in Supplementary eTable 2.

**Figure 2:**
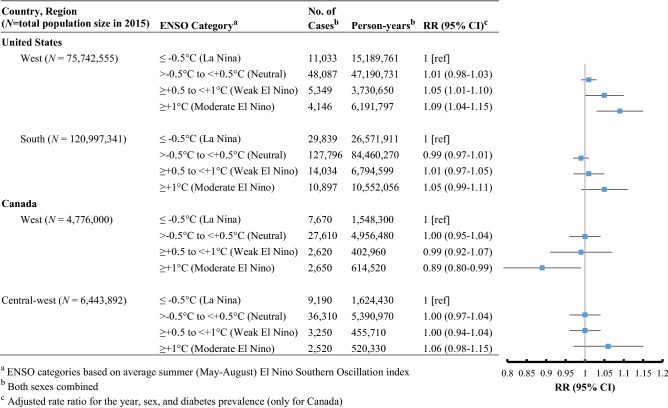
Association between ENSO categories and acute myocardial infarction-related mortality (United States) and admission (Canada) rates for older adults, 2000–2015.

Figure 2 shows the association between summer ENSO and AMI mortality and admission rates among older adults, controlling for year, sex, and diabetes prevalence (for Canadian analyses only). A summer moderate El Niño event was associated with a significantly reduced rate of AMI admission compared to summer years of La Niña events among older adults residing in West Canada (adjusted RR 0.89, 95% CI, 0.80–0.99), and an increased rate of AMI mortality among older adults in West United States (adjusted RR 1.09, 95% CI, 1.04–1.15). Similar analyses did not reveal a significant association between ENSO and AMI outcomes in the South United States or Central-west Canada. Findings were similar in regards to the occurrence of a weak El Niño during summertime for West United States only, during which there was a significant increase in AMI mortality rate among older adults compared to summer years of La Niña events (adjusted RR 1.05, 95% CI, 1.01–1.10). This association was not observed in West Canada (adjusted RR 0.99; 95% CI, 0.92–1.07). A sensitivity analysis that also adjusted for annual smoking and obesity rates resulted in similar findings (West Canada only; data not shown).

**Figure 3:**
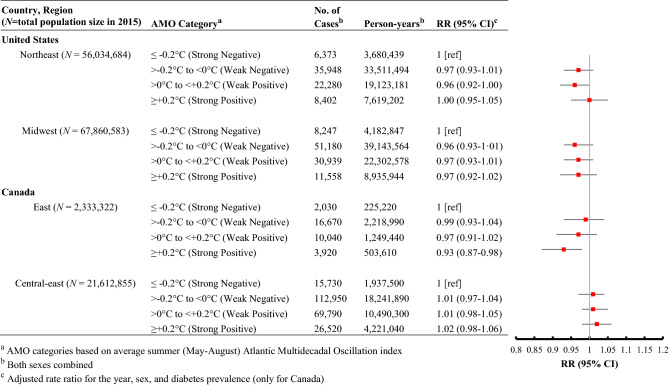
Association between AMO categories and acute myocardial infarction-related mortality (United States) and admission (Canada) rates for older adults, 2000–2015.

As depicted in Fig. 3, a strong positive phase of summer AMO was associated with a reduced rate of AMI admission among older adults in East Canada relative to years when the summer AMO was strongly negative (adjusted RR 0.93, 95% CI, 0.87–0.98). Extending our analysis to older adults in the United Kingdom (Table 1), we found that a strong positive phase of AMO during summer was associated with a significantly increased IHD mortality rate in the same months relative to summers with a weak negative phase of AMO, after controlling for sex and year (adjusted RR 1.08; 95% CI, 1.03–1.14). In contrast, summer ENSO was not significantly related to IHD mortality in the United Kingdom.

**Table 1:** Association between summer Atlantic Multidecadal Oscillation (AMO) or El Niño Southern Oscillation (ENSO) categories and ischemic heart disease mortality rates among older adults in the United Kingdom, 2010–2017.

Climate pattern^a^	No. of Cases^b^	Person-years^b^	RR (95% CI)^c^
**AMO**			
> − 0.2 °C to < 0 °C (Weak Negative)	13,552	21,381,113	1 [ref]
> 0 °C to < + 0.2 °C (Weak Positive)	10,096	16,780,413	1.03 (0.99–1.06)
≥ + 0.2 °C (Strong Positive)	3689	4,787,516	1.08 (1.03–1.14)
**ENSO**			
≤ − 0.5 °C (La Niña)	3387	5,765,980	1 [ref]
> − 0.5 °C to < + 0.5 °C (Neutral)	17,230	26,388,580	0.98 (0.91–1.06)
≥ + 0.5 to < + 1 °C (Weak El Niño)	3405	5,162,630	0.95 (0.86–1.05)
≥ + 1 °C (Moderate El Niño)	3315	5,631,852	0.96 (0.88–1.05)

^a^AMO and ENSO were run as separate models (i.e. not controlled for each other).

^b^Both sexes combined.

^c^Rate ratio after adjusted for the year and sex.

## Discussion

To our knowledge this is the first multi-national study investigating the association of large-scale climate patterns arising from SST variability on the risk of IHD outcomes among older adults. Moderate strength El Niño events create warm wet summers in West United States^19,21^, which we found evidence of association with an increased AMI mortality rate, whereas in West Canada this phase of ENSO leads to dry warm conditions^25^, and was associated with reduced AMI admission rates among older populations. In West Canada, a summer La Niña (i.e. cooler Pacific SST—creating cool wet summers)^25^ was associated with the highest rate of AMI admission. We also found an inverse association between summer AMO and AMI admission rates in East Canada, with a strong positive AMO (creating warm dry summers) conferring the lowest risk. In contrast, there was a direct relationship between AMO and IHD mortality during summers in the United Kingdom. A positive phase of summer AMO not only creates warmer SAT over the United Kingdom^23^, but also forces summers to be longer, which can lead to extended durations of summer cloud cover and precipitation over northwestern Europe^21–24^.

These findings suggest a role for large-scale climate patterns as a possible risk factor for IHD-related admissions/mortality. Common climatological conditions that were associated with an increased rate of IHD events were greater cloud cover/precipitation in the affected region. Most ecological studies have concentrated solely on a single climatological variable, where results have been inconsistent, with some authors reporting an association between extreme SAT and the likelihood of AMI^3–7^, whereas others suggesting that moderate SAT result in a greater burden^9,10^. A study reported up to 12% significant increase in rate of AMI admission among adults aged ≥ 70 years in some regions of California during El Niño events^16^. In addition, a smaller study in the Democratic Republic of the Congo noted a positive association between ENSO, increases in rainfall, and incidence of hemorrhagic strokes^17^.

There are some plausible biological mechanisms to explain why we found warmer and wetter summers to be associated with an increase in IHD event rates. For instance, extreme heat and humidity can lead to intravascular volume depletion and dehydration among susceptible populations. This is far more common among older adults, because they exhibit low subjective levels of thirst, and therefore may experience decreased plasma volume and blood pressure, as well as metabolic disturbances from excess fluid losses in response to extreme heat and inadequate levels of water intake^11^. Ultimately this cascade of events can cause reductions in coronary blood flow, thereby increasing the risk of an IHD event^29^. Dark/gloomy conditions could result in isolation and higher levels of stress and depression among older adults^30^. Physical activity levels in older adults are more pronounced during boreal summers in comparison to winters of northern countries^31^, though in some instances excess cloud cover and rainy conditions can deter physical activity, which may cause older individuals to be dormant indoors^32,33^. These conditions can also disrupt circadian phase shifts, affecting the timing and peak of cortisol concentrations^34^ and altering nutrient signaling, glucose regulation, blood pressure control, endothelial function and a host of other biological processes^35,36^. Disruption in circadian rhythms also reduces the duration and quality of sleep among older adults who already experience reduced quantities of deep sleep due to their age^37^, and has been demonstrated to predict the risk of cardiovascular disease^38^. A recent study documents warm summers can also lead to sleep disturbances among older adults, which can also increase the risk of IHD^13^.

Strengths related to this study include the use of population-based data which reduces the likelihood of selection bias, and these data sources captured IHD outcomes using well validated, and standardized definitions. However, there are a number of limitations that merit mentioning. Since the study’s approach was ecological, the attribution of causation to SST climate patterns as a risk factor for IHD outcomes cannot be met. United States and Canadian data on AMI mortality and admissions was only available on an annual basis, possibly underestimating the effect of such summer climate patterns on the risk of AMI during the same months. Also we were unable to examine the biological factors that mediate the relationship observed. Perhaps there could be other secular trends that confound the association between ENSO/AMO and IHD; hitherto our sensitivity analyses undertaken for western Canada, in which we adjusted for annual trends in smoking and obesity, did not appreciably change our results. Lastly, due to its periodicity there were only a few El Niño and La Niña events that occurred during the studied time frame.

## Conclusion

Our findings suggest that SST variability may have important consequences on the incidence and survival following IHD among older populations. While our study focused on natural climate variability, rising greenhouse gases are more likely to result in extreme ENSO events^39^. Anthropogenic climate change expected over the coming decades, may have dire consequences for IHD risk among older adults living in North America and the United Kingdom. Moreover, the importance of this study suggests that Pacific and Atlantic SST variability could be used to predict short-term changes in IHD event rates for older adults residing in North America and western Europe. For example, the magnitude of the ENSO index at the beginning of a year or season could be used to estimate the future burden of IHD in susceptible areas; which is more difficult to do based on a single climatological variable. Thus findings from this research could help alert public health professionals and acute care services as to when there may be an increase in IHD events so that preventative messaging and other mitigation strategies could be deployed upon an ENSO/AMO season. Therefore, this research highlights the growing need for research to understand the effects of climate change and climate variability on cardiovascular disease risk. Future studies are needed to better understand the influence of large-scale climate patterns on other cardiovascular diseases worldwide.

### Methods

This study used large climate and national health databases from the United States, Canada, and United Kingdom to examine the association between SST related climate patterns (ENSO/AMO) and IHD outcomes from 2000–2017. The specific IHD outcomes analyzed were based on the data availability and reporting preferences in each country. We restricted our analysis to older adults (≥ 65 years of age) who are likely to be more susceptible to climatic changes than young and mid-aged populations^8^. Average ENSO and AMO indices were derived from May to August. In contrast, IHD outcomes were reported as annual rates because of limited availability of monthly or seasonal data, with the exception of data from the United Kingdom.

### National clinical data registries

For the United States, the annual number of deaths due to AMI among older adults (65–74 years of age) were acquired from the Centers for Disease Control and Prevention (CDC) WONDER database from 2000–2015. Deaths due to AMI were based on the International Classification of Diseases (ICD) 10th revision (ICD-10) codes (I21 and I22) listed in individual records. Annual AMI mortality rates (number of deaths from AMI per 100,000 population) for older adults were ascertained by sex and United States census bureau-designated regions (West, South, Midwest, and Northeast) using CDC data for the numerator and population counts from the United States census in the same year as the denominator^40^.

The Canadian Chronic Disease Surveillance System (CCDSS) reports information on the incidence and prevalence of many chronic conditions at the national and provincial level. CCDSS data were used to ascertain the annual number of incident hospital admissions for AMI in each Canadian province from 2000–2015 among older adults (65–79 years of age)^41^. Data from hospital discharge abstracts were initially collected by the Canadian Institute for Health Information and those meeting the diagnostic criteria for AMI were coded using an ICD-9 code of 410 (for records prior to April 2002) or ICD-10 code of I21 or I22 (for records from April 2002 onwards). Annual admission rates (number of incident AMI cases per 100,000 population) were calculated by sex and region [West (British Columbia), Central-west (Alberta, Saskatchewan, and Manitoba), Central-east (Ontario and Quebec), and East (New Brunswick, Nova Scotia, Prince Edward Island, and Newfoundland and Labrador)] using counts from CCDSS and population denominators from the Canadian census (to the nearest census year). Annual sex and region-specific diabetes prevalence rates for older adults were also ascertained from the CCDSS for inclusion in our models.

For the United Kingdom, monthly data on the number of IHD-related deaths among older adults (65–74 years of age) in England and Wales were acquired from the Office for National Statistics (ONS, reference #:009367) throughout January 2010 to December 2017. Numerators were based on the presence of one or more ICD-10 codes for IHD (I20-25) listed on each death record in a given year, with denominators based on mid-year population estimates based on age and sex. Summer IHD mortality rates were computed by sex based on deaths occurring each year between May and August, and reported as the number of deaths per 100,000 population among men and women.

### Climatological/exposure data

One of our primary exposures involved the ENSO index which reflects deviations in SST over the central equatorial Pacific region, referred to as the Niño 3.4 region (5 N–5S, 170 W–120 W)^26^. Similarly, the AMO index is defined by deviations in SST over the North Atlantic basin (0°N–70°N and 75°W–7°W)^23^. Average monthly ENSO and AMO indices were obtained for each year throughout 2000–2017 from the National Oceanic and Atmospheric Administration (NOAA)^42^. As Pacific SST warm towards the summer, the evolution of an ENSO phase typically begins in boreal spring/summer^26^. The climatological impact that AMO has over western Europe is strong during the summer^23^, for this reason average summer ENSO and AMO indices from May to August of each year were computed. We then derived a measure of interannual variability that reflected the observed deviation in summer ENSO and AMO from that expected for a given year^43^. To do so, we fit separate simple linear regression models on summer ENSO and AMO index by year. The interannual variability in summer ENSO and AMO indices were constructed by subtracting the observed by expected value for a given year and was plotted from 2000–2017 (Supplementary eFigure 1).

For each year, the interannual variability in summer ENSO index (referred herein as ENSO variability) was classified into four categories from cooler to warmer values; ≤  − 0.5 °C (La Niña), >  − 0.5 °C to <  + 0.5 °C (neutral), ≥  + 0.5 °C to <  + 1 °C (weak El Niño), and ≥  + 1 °C (moderate El Niño)^26^. Similarly, AMO variability was classified as; ≤  − 0.2 °C (strong negative), >  − 0.2 °C to < 0 °C (weak negative), > 0 °C to <  + 0.2 °C (weak positive), and ≥  + 0.2 °C (strong positive).

Geospatial data of SST, SAT, and cloud cover data were acquired by the European Centre for Medium-Range Weather Forecasts (ECMWF), ERA5, which provides climatological records based on a combination of modern atmosphere assimilation systems and historical data^44^.

### Descriptive analysis

We derived annual rates of AMI/IHD admissions or deaths among older adults according to ENSO/AMO categories, sex and region, by summing both numerators (counts of admissions/deaths) in a given year affected by each ENSO/AMO category and population denominators during those same years. All rates were reported as the number cases per 100,000 person-years. We also generated geospatial summer anomalies of average summer SST, SAT and cloud cover based on deviations from the conventional summer climatology period 1981–2010 for three chosen years of positive and negative ENSO and AMO events^19^. This way, each set of maps illustrated summertime anomalies during positive or negative ENSO and AMO relative to the reference period.

### Statistical analysis

We examined the association between summer ENSO variability and AMI-related mortality (United States) and incidence AMI admission (Canada) rates in regions adjacent to the Pacific Ocean (West United States/Canada) where a summer ENSO event is known to have a strong climatological impact, and a comparison region (South United States/Central-west Canada), where the effect of ENSO would be expected to be less. To address the presence of serial autocorrelation (the tendency for rates in the prior and subsequent year to be highly correlated)^43^, we used negative binomial regression with autocorrelated residuals of order two, adjusting for sex, diabetes prevalence (only for Canada), and secular trend (i.e. year). AMI admission/mortality rates were log transformed, so the estimated parameters were interpreted as a rate ratio (RR). The model is described in the equation below:

$$ \log \left( {AMI} \right)_{t} = {\text{intercept}} + \beta_{1} \left( {ENSO/AMO} \right)_{t} + \beta_{2} \left( {Year} \right)_{t} + \beta_{3} \left( {Sex} \right)_{t} + \beta_{4} \left( {Diabetes} \right)_{t} + \varepsilon_{t} $$

where $$\varepsilon_{t} = \varphi_{1} \varepsilon_{t - 1} + \varphi_{2} \varepsilon_{t - 2} + \alpha_{t} ,  \;\alpha_{t} \sim N\left( {0, \sigma^{2} } \right)$$.

We used a similar approach to study the association between summer AMO categories and AMI mortality and admission rates in affected regions in the United States (Northeast/Midwest) and Canada (East/Central-east), based on the known influence of Atlantic SST variability on eastern North America climatology.

For analyses involving United Kingdom data, we examined the association between summer ENSO/AMO categories (separately) and summer IHD mortality rates, while controlling for year and sex. These associations were also conducted using negative binomial regression with autocorrelative residuals of order one,

$$ \log \left( {IHD} \right)_{t} = {\text{intercept}} + \beta_{1} \left( {ENSO/AMO} \right)_{t} + \beta_{2} \left( {Year} \right)_{t} + \beta_{3} \left( {Sex} \right)_{t} + \varepsilon_{t} $$

where $$\varepsilon_{t} = \varphi_{1} \varepsilon_{t - 1} + \alpha_{t} ,\;\alpha_{t} \sim N\left( {0, \sigma^{2} } \right)$$.

As an additional sensitivity analysis, we adjusted our models for the annual prevalence of smoking and obesity among older adults residing in West Canada (i.e. British Columbia). Yet, there was no meaningful change in RR or their 95% CI upon assessing the association between ENSO/AMO on AMI when we included the latter covariates in the model (data not shown). All computation, analyses, and figure creation used a combination of Microsoft Excel 2013, RStudio, and Matlab R2017b.

## Supplementary Information

Supplementary Information 1.

## Data Availability

The authors declare that all data supporting the findings of this study are available within this paper and its Supplementary Information files.
