# Supplementary material for: Sea surface temperature variability and ischemic heart disease outcomes among older adults
Source: Sci Rep. 2021 Feb 9;11:3402. doi: 10.1038/s41598-021-83062-x (PMC7873280; doi:10.1038/s41598-021-83062-x)
Supplement: Supplementary file 1 — Supplementary Information 1. [file 41598_2021_83062_MOESM1_ESM.pdf]

Supplementary Online Content

Sea surface temperature variability and ischemic heart disease outcomes among older adults

Haris Majeed[1,2]*, Rahim Moineddin[3,4], Gillian L. Booth[1,2,4,5]

[1] Institute of Medical Science, Temerty Faculty of Medicine, University of Toronto, 1 King's College Circle, Toronto, Ontario, Canada, M5S 1A8

[2] MAP Centre for Urban Health Solutions, Li Ka Shing Knowledge Institute of St Michael's Hospital, Unity Health Toronto, 209 Victoria Street, Toronto, Ontario, Canada M5B 1T8

[3] Department of Family and Community Medicine, Temerty Faculty of Medicine, University of Toronto, 500 University Avenue, Toronto, Ontario, Canada M5G 1V7

[4] Institute of Health Policy, Management and Evaluation, Dalla Lana School of Public Heath, University of Toronto, 155 College Street, Toronto, Ontario, Canada M5T 1P8

[5] Department of Medicine, Temerty Faculty of Medicine, University of Toronto, 1 King's College Circle, Toronto, Ontario, Canada, M5S 1A8

*Author to whom correspondence should be addressed: haris.majeed@utoronto.ca

Supplementary eTable 1. Sex-specific ischemic heart disease event rates among older adults in the United States, Canada, and United Kingdom.

	**Men**						**Women**					
	**Start of Study (2000)**			**End of Study (2015)**			**Start of Study (2000)**			**End of Study (2015)**		
**Condition**	Cases	Population	Rate[a]	Cases	Population	Rate[a]	Cases	Population	Rate[a]	Cases	Population	Rate[a]
AMI Mortality												
West United States	3,680	1,688,412	218.0	2,744	2,928,262	93.7	2,137	1,973,866	108.3	1,402	3,263,535	43.0
South United States	9,631	3,022,068	318.7	6,997	4,910,840	142.5	6,080	3,685,925	165.0	3,900	5,641,216	69.1
Midwest United States	5,970	1,918,250	311.2	3,713	2,753,567	134.8	3,546	2,327,349	152.4	1,912	3,084,387	62.0
Northeast United States	4,542	1,671,240	271.8	2,262	2,285,149	99.0	4,873	2,096,781	137.0	1,257	2,660,066	47.3
AMI Admission												
West Canada	1,720	187,020	919.7	1,770	292,880	604.3	970	210,350	461.1	880	321,640	273.6
Central-west Canada	2,230	210,870	1057.5	1,560	246,970	631.7	1,240	243,490	509.3	960	273,360	351.2
Central-east Canada	9,940	858,830	1157.4	8,820	1,258,430	700.9	6,510	1,055,070	617.0	5,210	1,457,550	357.4
East Canada	1,300	101,970	1274.9	1,390	160,660	865.2	840	122,720	684.5	790	180,480	437.7
	**Start of Study (2010)**			**End of Study (2017)**			**Start of Study (2010)**			**End of Study (2017)**		
IHD Mortality	Cases	Population	Rate[a]	Cases	Population	Rate[a]	Cases	Population	Rate[a]	Cases	Population	Rate[a]
United Kingdom[b]	2,621	2,298,337	342.2	2,403	2,819,298	255.7	1,069	2,489,179	128.8	901	3,032,505	89.1

[a] Rate reported as per 100,000 person-years

[b] England and Wales ischemic heart disease deaths between May-August

Supplementary eTable 2. Expected climatological conditions during summer months (May-August) in United States and Canada during El Niño Southern Oscillation (ENSO) or Atlantic Multidecadal Oscillation (AMO) phases.

**Affected Regions**	**El Niño Southern Oscillation**[a]		**Atlantic Multidecadal Oscillation**[a]	
	Positive (El Niño)[b]	Negative (La Niña)[b]	Positive[c]	Negative[c]
United States				
West	warm and wet	cool and dry		
South	cool and wet	warm and dry		
Midwest			warm and dry	cool and wet
Northeast			warm and dry	cool and wet
Canada				
West	warm and dry	cool and wet		
Central-west	warm	cool		
Central-east			warm	cool
East			warm and dry	cool

[a] Summer conditions along with their intensity may vary for each state/province, depending on the strength of ENSO/AMO

[b] ENSO index ≥+0.5°C (El Niño) whereas ≤-0.5°C (La Niña)

[c] AMO index >0°C (positive) whereas <0°C (negative)

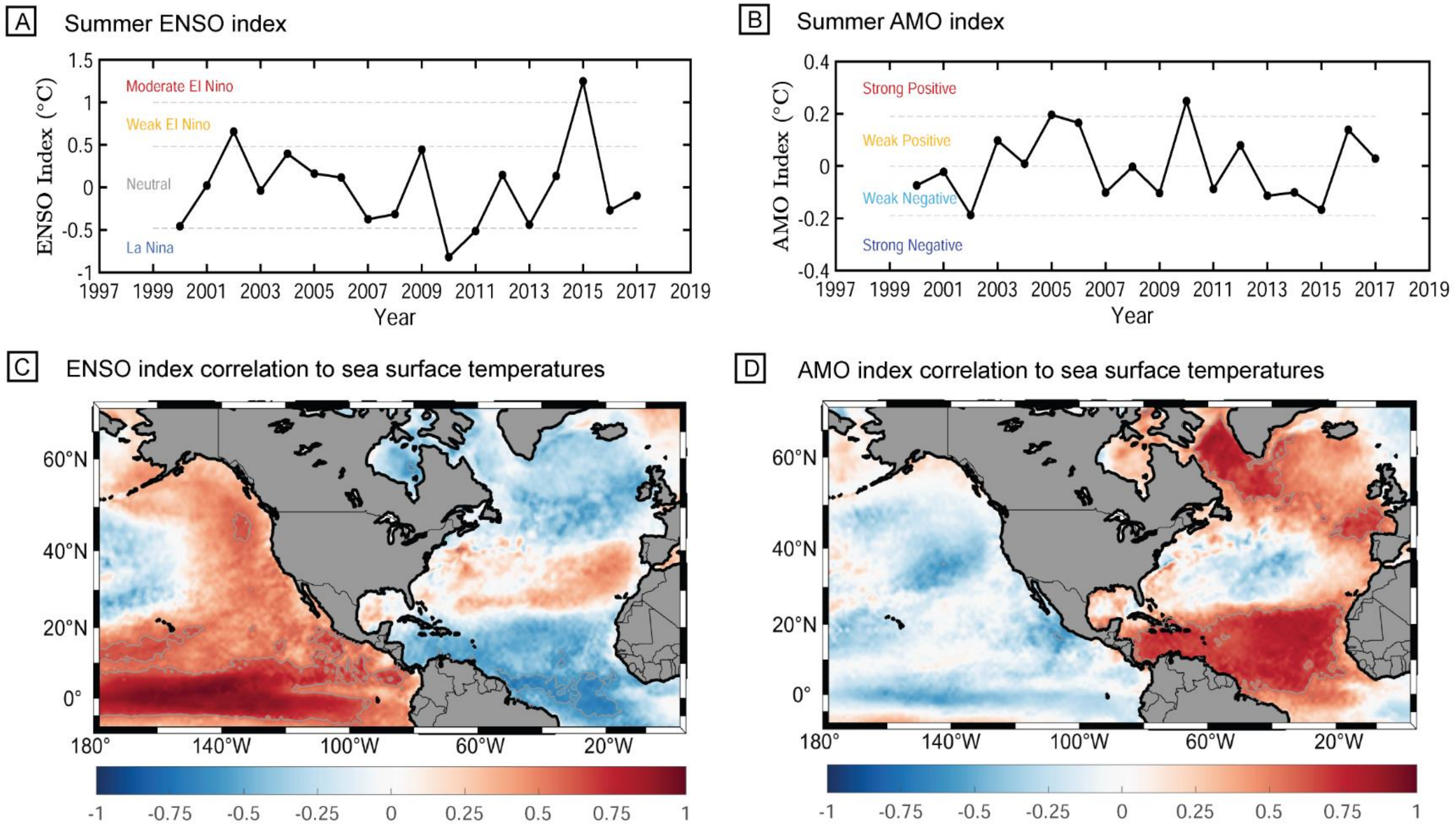

Supplementary eFigure 1. Summer ENSO and AMO indices and geospatial SST patterns, 2000-2017.
(A) Interannual variability in average summer ENSO and (B) AMO index, 2000-2017. (C) Geospatial grid point correlation of sea surface temperature to this ENSO index and (D) AMO index, 2000-2017. Regions with statistically significant (P≤0.05) correlations have been contoured grey.
